# A Novel Interaction between a 23-SNP Genetic Risk Score and Monounsaturated Fatty Acid Intake on HbA1c Levels in Southeast Asian Women

**DOI:** 10.3390/nu16173022

**Published:** 2024-09-06

**Authors:** Padmini Sekar, Arif S. Aji, Utami Ariyasra, Sri R. Sari, Nabila Tasrif, Finny F. Yani, Julie A. Lovegrove, Ikhwan R. Sudji, Nur I. Lipoeto, Karani S. Vimaleswaran

**Affiliations:** 1 Hugh Sinclair Unit of Human Nutrition, Department of Food and Nutritional Sciences, Institute for Cardiovascular and Metabolic Research (ICMR), University of Reading, Reading RG6 6DZ, UK; p.sekar@pgr.reading.ac.uk (P.S.); j.a.lovegrove@reading.ac.uk (J.A.L.); 2 Department of Nutrition, Faculty of Health Sciences, Alma Ata University, Bantul, Yogyakarta 55183, Indonesia; sabtaaji@almaata.ac.id; 3 Department of Biomedical Science, Faculty of Medicine, Andalas University, Padang 25163, Indonesia; tamiariyasra13@gmail.com (U.A.);; 4 Culinary Study Program, Faculty of Tourism and Hospitality, Universitas Negeri Padang, Padang 25163, Indonesia; tasrifnabila@unp.ac.id; 5 Department of Child Health, Faculty of Medicine, Andalas University, Padang 25163, Indonesia; finny_fy@yahoo.com; 6 Biomedical Laboratory, Faculty of Medicine, Andalas University, Padang 25163, Indonesia; ir.sudji@gmail.com; 7 Department of Nutrition, Faculty of Medicine, Andalas University, Padang 25163, Indonesia; indra.liputo@gmail.com; 8 Institute for Food, Nutrition and Health (IFNH), University of Reading, Reading RG6 6AH, UK

**Keywords:** Minangkabau women, Indonesia, genetic risk score, gene–diet interaction, single nucleotide polymorphism, metabolic diseases

## Abstract

Metabolic diseases result from interactions between genetic and lifestyle factors. Understanding the combined influences of single-nucleotide polymorphisms (SNPs) and lifestyle is crucial. This study employs genetic risk scores (GRS) to assess SNPs, providing insight beyond single gene/SNP studies by revealing synergistic effects. Here, we aim to investigate the association of a 23-SNP GRS with metabolic disease-related traits (obesity and type 2 diabetes) to understand if these associations are altered by lifestyle/dietary factors. For this study, 106 Minangkabau women were included and underwent physical, anthropometric, biochemical, dietary and genetic evaluations. The interaction of GRS with lifestyle factors was analyzed using linear regression models, adjusting for potential confounders. No statistically significant associations were observed between GRS and metabolic traits; however, this study demonstrates a novel interaction observed between 13-SNP GRS and monounsaturated fatty acid (MUFA) intake, and that it had an effect on HbA1c levels (*p* = 0.026). Minangkabau women with low MUFA intake (≤7.0 g/day) and >13 risk alleles had significantly higher HbA1c levels (*p* = 0.010). This finding has implications for public health, suggesting the need for large-scale studies to confirm our results before implementing dietary interventions in the Indonesian population. Identifying genetic influences on dietary response can inform personalized nutrition strategies to reduce the risk of metabolic disease.

## 1. Introduction

Metabolic diseases, including obesity and type 2 diabetes (T2D), are a leading cause of global mortality, claiming 17.9 million lives annually [1]. Behavioral risk factors such as physical inactivity, tobacco use, an unhealthy diet and alcohol consumption contribute to increased blood glucose levels, and overweight/obesity [2]. With over 1 billion people worldwide suffering from obesity, the World Health Organization (WHO) projects that by 2025, 167 million individuals are at risk of facing health issues due to being overweight or obese [3]. The global incidence of T2D has also increased, with 1.5 million deaths annually directly linked to diabetes, mostly T2D [4]. Across the world, approximately 422 million individuals are affected majorly by T2D, with a high incidence in low- and middle-income countries (LMICs), like those in Southeast Asia [5].

Indonesia, categorized as a LMIC with a population exceeding 280 million people, is witnessing a rise in childhood obesity and overweight, as commonly seen in many LMICs [6]. According to recent 2023 reports, Indonesia is among the top 10 countries globally with a substantial prevalence of T2D, at 10.8% [6]. The country is home to over 50 ethnic groups, with the Minangkabau women of West Sumatra forming the world’s largest matrilineal society. Research focusing on the Minangkabau women has revealed poor dietary fat patterns, characterized by unhealthy fat sources (such as processed food and meat, fast food, and deep-fried foods) and a high risk of obesity and T2D [7,8]. This highlights the urgent need for further research on the dietary patterns within the Minangkabau community, particularly among women, to better understand and address the increasing rates of metabolic diseases. We hypothesize that the interaction between a novel 23-SNP metabolic genetic risk score (GRS) chosen for this study and dietary/lifestyle factors significantly influences metabolic traits, including HbA1c levels, in Minangkabau Indonesian women. This interaction may reveal specific genetic and dietary patterns that contribute to the observed metabolic outcomes in this population. 

Furthermore, it is important to evaluate the genetic factors involved in the onset and development of metabolic diseases in Minangkabau women. A recent systematic review from our team identified 29 significant gene–diet interactions impacting the risk of diabetes and obesity in Southeast Asian populations [9]. The systematic review also identified six studies showing gene–diet interactions impacting obesity-related traits [10,11,12,13,14,15] and three studies impacting diabetes-related outcomes [10,11,13] in the Indonesian population. The metabolic traits are influenced by numerous single-nucleotide polymorphisms (SNPs), each exerting a relatively modest impact on the trait in isolation [16]. Examining the cumulative effects of multiple SNPs could offer a more comprehensive understanding of individual trait variability and enhance the predictive accuracy for metabolic diseases, surpassing the limitations of a single-variant approach [17]. It is certain that employing the GRS approach enhances our ability to identify gene–lifestyle interactions, surpassing the effectiveness of conventional single SNP methods [18]. Hence, the current study aims to investigate the interaction between a novel 23-SNP metabolic GRS and dietary factors on metabolic traits (obesity and T2D) in 106 Minangkabau Indonesian women.

## 2. Materials and Methods

### 2.1. Study Participants

This study included healthy women registered in the Minangkabau Indonesia Study on Nutrition and Genetics (MINANG). This cross-sectional pilot study took place in Padang, West Sumatra, Indonesia, between December 2017 and January 2018. The MINANG study is an integral component of the ongoing GeNuIne (Gene-Nutrient Interactions) Collaboration [19,20,21,22,23] that explores the interplay between dietary factors and genetics (nutrigenetics) concerning cardiometabolic disease and its associated traits from different ethnic groups [23]. The detailed methodology for this study is available elsewhere [13]. In brief, the study recruited 133 women from public health centers in Puskesmas Andalas and Puskesmas Kuranji, representing the urban and rural populations of Padang, respectively. The inclusion criteria comprised healthy women aged 25–60 (mean age = 40.53 ± 10.18) who belonged to the Minangkabau ethnicity. Among the initially enrolled participants, 16 were excluded due to factors such as pregnancy or lactation, a history of metabolic or communicable diseases, supplement intake, or being a relative of another participant. For this study, another 11 participants were excluded due to incomplete dietary information (*n* = 2) and genotype data (*n* = 9) from the genetic risk score. The final sample comprised 106 participants. The MINANG study adhered to the principles outlined in the Declaration of Helsinki and received approval from the Ethical Review Committee of the Medical Faculty, Andalas University (No.311/KEP/FK/2017). Prior to participation, all individuals provided written informed consent and retained the right to withdraw from the study at their discretion, opting out of any procedures.

### 2.2. Anthropometry Measurements

The participants’ body weight (to the nearest 100 g) and height (to the nearest mm) were measured using an electronic scale (Seca 803, Seca GmbH. Co. kg, Hamburg, Germany) and a wall-mounted stadiometer (OneMed Medicom stature meter, YF.05.05. V.A.1022, Indonesia), respectively. Their body mass index (BMI) was calculated by dividing their weight (in kg) by the square of their height (in m^2^), with the obesity classification determined according to the Asia-Pacific criteria [24]. Their body fat percentage (BFP) was measured using the Tanita MC780 (TANITA, Tokyo, Japan) multi-frequency segmental body composition analyzer. Their waist circumference (WC) was measured in cm using a metal tape (Medline-OneMed Medicom, Jakarta, Indonesia) positioned midway between the 12th rib and the superior border of the iliac crest, with measurements taken at the end of a normal expiration.

### 2.3. Biochemical and Clinical Measures

Fasting blood samples (5 mL) were collected by a trained phlebotomist, with serum separated and stored at −20 °C until further analysis. After a 12 h fasting period, blood samples (5 mL) were taken to measure the concentrations of glucose, insulin, glycated hemoglobin A1c (HbA1c), total cholesterol, triglycerides, low-density lipoprotein-c (LDL-c), and high-density lipoprotein-c (HDL-c). Sample analysis was performed using the xMark Microplate Spectrophotometer (Bio-Rad Laboratories Inc., Hercules, CA, USA). Fasting glucose, insulin, and HbA1c levels were determined using enzyme-linked immunosorbent assay (ELISA) kits from Bioassay Technology Laboratory (Shanghai, China). The participants’ fasting blood lipid levels were assessed through enzymatic colorimetric methods, with triglycerides measured by the glycerine phosphate oxidase peroxidase (GPO-PAP) method and total cholesterol and HDL-c quantified using the cholesterol oxidase phenol 4-aminoantipyrine peroxidase (CHOD-PAP) technique. The concentration of LDL-c was calculated using Friedewald’s formula. Their systolic and diastolic blood pressures (SBP and DBP) were measured using a sphygmomanometer, with two readings taken at 5 min intervals, and the average recorded.

### 2.4. Assessment of Dietary Intake

An experienced nutritionist gathered data regarding dietary intake and physical activity either at participants’ homes or in an integrated health service post. Dietary habits were evaluated through a validated, published semi-quantitative food frequency questionnaire (SQ-FFQ) with a comprehensive list of 223 food items [25]. The collected data underwent a thorough double-check to ensure accuracy and were subjected to analysis using the Indonesian Food Database and Nutrisurvey (EBISpro, Willstätt, Germany, https://www.ebispro.de/, accessed on 21 July 2024). This analysis aimed to evaluate the overall energy and macronutrient intake. The nutrient intake values were adjusted for total energy intake using the nutrient (energy-adjusted) residual method, as deemed suitable [26].

### 2.5. Assessment of Physical Activity

A proficient nutritionist gathered data regarding the assessment of the participants’ physical activity level; this included transport, work, and leisure time, as well as time spent on sedentary behavior, and was conducted using “The Global Physical Activity Questionnaire” (GPAQ) [27]. The total duration of moderate-to-vigorous physical activity was calculated following the World Health Organization (WHO) STEP-wise method and calculated as metabolic equivalent minutes per day (METmins/day). Participants were categorized as “active” if they achieved ≥ 600 METmins/week or “inactive” if their accumulation was < 600 METmins/week.

### 2.6. SNP Selection and Genotyping

Twenty-three genetic variants located on 19 different genes were shortlisted for this study on the basis of a consistent association with metabolic traits from previous studies [28,29,30,31,32,33,34,35,36,37,38,39,40,41,42,43,44,45]. The selected genetic variants were Methylenetetrahydrofolate reductase (*MTHFR*) rs1801133 [28,46], Transcription factor 7 like 2 (*TCF7L2*) SNPs rs7903146 [29] and 12255372 [32], the fat mass and obesity-associated (*FTO*) SNPs rs8050136 [47] and 9939609 [46], Melanocortin-4 receptor (*MC4R*) rs17782313 [31], Peroxisome proliferator-activated receptor gamma (*PPARG*) rs1801282 [33], Potassium inwardly rectifying channel, subfamily J, member 11 protein (*KCNJ11*) rs5219 [28], Potassium Voltage-Gated Channel Subfamily Q Member 1 (*KCNQ1*) SNPs rs2237895 [48] and 2237892 [34], Cytochrome P450 Family 2 Subfamily R Member 1 (*CYP2R1*) SNPs rs10741567 and rs12794714 [37], NAD synthetase 1 (*NADSYN1*) rs12785878 [36,49], Cytochrome P450 family 24 subfamily A member 1 (*CYP24A1*) rs6013897 [49], GC vitamin D-binding protein (*GC*) rs2282679 [36], DAB adaptor protein 1 (*DAB1*) rs6680429 [38], Adiponectin, C1Q and collagen domain containing (*ADIPOQ*) rs266729 [39], Cyclin Dependent Kinase Inhibitor 2A (*CDKN2A/B*) rs10811661 [40], Calcium sensing receptor (*CASR*) rs1801725 [41], Calpain 10 (*CAPN10*) rs5030952 [42], ATP binding cassette subfamily D member 4 (*ABCD4*) rs3742801 [43], Metabolism of cobalamin-associated A (*MMAA*) rs2270655 [44], Fucosyltransferase 6 (*FUT6*) rs778805. The 23 SNPs that were chosen for this study were checked for Hardy–Weinberg Equilibrium (*p* > 0.05) (Supplementary Table S1), as determined by the goodness-of-fit chi-square test.

Genomic DNA was extracted from peripheral blood leukocytes using the PureLink Genomic DNA Mini Kit (Invitrogen, Carlsbad, CA, HQ USA). The DNA concentration was then determined using a NanoDrop spectrophotometer. The SNPs were genotyped through the competitive allele-specific PCR-KASP^®^ assay at LGC Genomics (http://www.lgcgroup.com/services/genotyping, accessed on 1 February 2024).

### 2.7. Statistical Analysis

Statistical analysis was conducted using SPSS software (version 28). The baseline characteristics of the continuous variables were expressed as means ± standard deviations (SD). Group comparisons were evaluated using one-way analysis of variance (ANOVA). The normality of continuous variables was tested and confirmed with the Shapiro–Wilk test. Log transformation was applied to variables not normally distributed, including age (years), body mass index (BMI) (kg/m^2^), waist circumference (WC) (cm), physical activity (min/week), fat mass (kg), glucose (mg/dL), cholesterol (mg/dL), HDL (mg/dL), LDL (mg/dL), triglycerides (TGL) (mg/dL), HbA1c (ng/mL), fasting insulin (ml/U), total energy (kcal), protein (g/day), fiber (g/day), fat (g/day), SFA (g/day), PUFA) (g/day and MUFA (g/day).

The 23-SNP GRS was constructed by summing the risk allele count for each SNP. Values of 0, 1, or 2 were assigned to each SNP, indicating the number of risk alleles for that SNP. For the GRS, the risk alleles were categorized into a “low-risk group” and “high-risk group” using a median value. In this study, individuals carrying ≤13 (*n* = 53) risk alleles were classified as “low risk”, while those carrying >13 (*n* = 53) risk alleles were grouped as “high risk”. The effect of the 23-SNP GRS on anthropometric and biochemical parameters was performed using linear regression models. The interactions between the 23-SNP GRS and dietary factors and their effect on anthropometric and biochemical parameters were tested using a general linear model, incorporating the (GRS*dietary factor) term in the model. The tests were adjusted for age, BMI (when BMI was not the outcome), and total energy intake, where applicable. The dietary factors considered for analysis included protein (g/day), fiber (g/day), fat (g/day), MUFAs (g/day), SFAs (g/day), and PUFAs (g/day). A *p*-value of <0.05 was considered statistically significant. Interactions that were statistically significant were further explored by categorizing participants based on tertiles of dietary intake.

## 3. Results

### 3.1. Characteristics of Participants Stratified Based on GRS

A total of 106 Minangkabau women aged between 25 and 60 years were included in this study. The genotype distribution of study participants is shown in Supplementary Table S1 and their characteristics based on GRS are presented in Table 1. Upon categorizing the women into the “low-risk group” (≤13 risk alleles; *n* = 53) and “high-risk group” (>13 risk alleles; *n* = 53), a significant difference was observed in the mean total energy intake (*p* = 0.040) between the two groups. However, there were no significant differences in the anthropometric and biochemical parameters between these groups (*p* > 0.05 for all comparisons).

### 3.2. Interactions between GRS and Lifestyle Factors on Anthropometric and Biochemical Parameters

**Figure 1:**
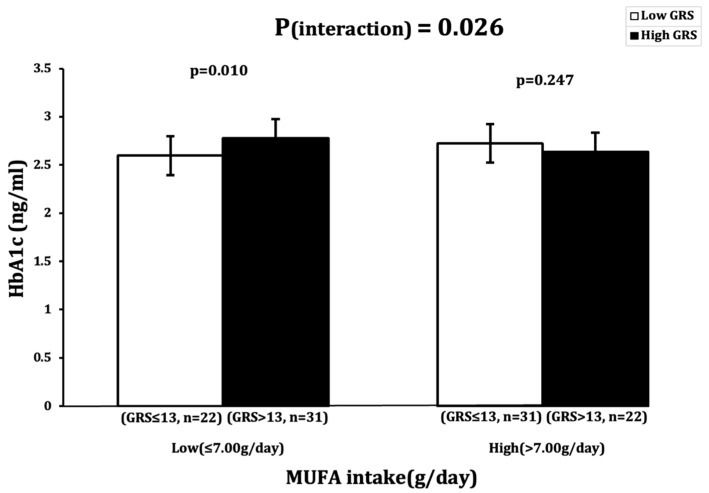
Interaction between 23SNP GRS and MUFA intake (g) and its impact on log-transformed HbA1c (ng/dl). White bars indicate “low genetic risk” group (≤13 risk alleles); black bars indicate “high genetic risk” group (>13 risk alleles). Carriers of 14 or more risk alleles with low MUFA intake (≤7.00 g) had higher HbA1c levels.

A significant interaction was observed between GRS and MUFA intake (g/day), with an impact on HbA1c (ng/mL) levels (P_interaction_ = 0.026) (Table 2). Participants with low MUFA intake (≤7.00 g) and a high GRS (>13 risk alleles) had significantly higher HbA1c levels (*p* = 0.010) than participants with low MUFA intake and a low GRS (≤13 risk alleles) (Figure 1).

## 4. Discussion

This study aimed to understand the interaction between a novel 23-SNP metabolic GRS and lifestyle factors, and its impact on anthropometric and biochemical parameters related to obesity and T2D in 106 Minangkabau women. Our study demonstrated that interactions between the 23-SNP GRS and MUFA intake had an impact on the HbA1c level, where individuals with low MUFA (≤7.0 g/day) intake and ≥13 risk alleles had significantly higher HbA1c levels compared to participants with less than 13 risk alleles. Given that higher levels of HbA1c serves as a risk predictor for T2D, these cross-sectional data suggest a possible link between MUFA intake and metabolic risk; however, these findings need to be confirmed in future studies. This could have a notable impact on public health by informing lifestyle intervention strategies for managing metabolic diseases such as T2D among Minangkabau women.

According to WHO recommendations, it is advised that one’s daily MUFA intake constitutes approximately 15% (30 g in a standard 2000 kcal diet) of one’s total energy intake [50]. Considering the average total energy intake of the participants in our study, their MUFA intake should have been around 54 g according to daily recommendations established by the WHO. However, they had a mean MUFA intake of only 8.3 g, significantly below the recommended level. Dietary imbalances, including a low intake of MUFA, have been linked to insulin resistance and cardiovascular risk factors in South Asians [51]. Previous studies have provided insights into a plausible molecular mechanism suggesting that a dietary regimen characterized by low levels of MUFA intake could potentially contribute to elevated HbA1c levels in patients with abnormal glucose metabolism [52]. Some genes selected for this study, such as *TCF7L2*, *FTO* (rs9939609 variant), and *CAPN10*, have been shown to be associated with diabetes-related traits such as HbA1c levels, when diets are low in MUFAs and high in SFAs [52,53]. *TCF7L2* has been strongly linked to elevated HbA1c levels and an increased risk of T2D [54], *FTO* rs9939609 variant has been linked to high HbA1c levels and insulin resistance [55,56], and *CAPN10* has been associated with insulin resistance and T2D [57]. However, further research is needed to establish direct links between these genes, cardiometabolic risk and low MUFA intake. 

MUFA-rich diets have been understood to reduce total and LDL cholesterol, and are more effective in reducing the risk of cardiovascular disease than low-fat, high-carbohydrate diets [52,58]. Furthermore, they can lead to a decrease in diastolic and systolic blood pressure, have a hypoglycemic effect and reduce HbA1c levels in T2D subjects [50]. Reports have shown that Minangkabau women have a dietary pattern with a higher intake of SFAs and a lower intake of MUFAs, which is linked to an increased risk of T2D and dyslipidemia [59,60]. In vivo studies have shown the potential molecular mechanisms by which MUFAs can play a defensive role in the progress of T2D. A study comparing the effects of MUFA- and SFA-rich diets on insulin resistance in rats reported that a MUFA-rich diet resulted in improved insulin sensitivity, but that the SFA-rich diet demonstrated the decreased movement of glucose transporter type 4 (*GLUT4*) to the skeletal muscle cell membrane, resulting in impaired insulin signaling. This highlights the positive effect of dietary MUFA on insulin sensitivity by preserving the IRS-1/PI3 kinase insulin signaling pathway, which, on the other hand, is disrupted by the SFA diet [61]. MUFAs have been shown to influence cell membrane fluidity, affecting insulin receptor function and facilitating *GLUT4* translocation to the cell membrane, further enhancing insulin-mediated cellular glucose uptake [62,63]. MUFA-rich diets have also been shown to enhance insulin sensitivity by activating *PPAR*-γ, which improves insulin receptor function and glucose uptake [62]. Additionally, MUFA improves mitochondrial function, essential for energy production and cellular metabolism, thereby enhancing insulin sensitivity and overall metabolic health [63]. Furthermore, a randomized clinical study showed that participants with high MUFA intake, such as those following the Mediterranean diet, showed significant improvements in adiponectin levels [64]. Adiponectin has anti-inflammatory and insulin-sensitizing properties, and high circulating levels have been associated with a lower risk of T2D and a reduction in the need for antihyperglycemic medications [65]. 

The integration of MUFA-enriched foods into dietary patterns has been recommended strongly, particularly for decreasing the risk of T2D [52] and offering a spectrum of additional health benefits [66]. Our findings support the development of personalized nutrition guidance, allowing clinicians to tailor dietary recommendations based on an individual’s genetic profile, particularly in populations with similar genetic backgrounds. Additionally, dietary interventions targeting MUFA intake could be prioritized in public health strategies aimed at preventing or managing T2D in genetically predisposed individuals. This study also underscores the potential for genetic screening programs to identify individuals at a higher risk of elevated HbA1c levels, enabling early intervention and reducing the incidence of T2D and related complications. Furthermore, the findings could inform public health policies in Indonesia and other Southeast Asian countries by emphasizing the incorporation of genetic information into dietary guidelines, leading to more effective prevention strategies for metabolic diseases. While the results are promising, larger-scale studies are recommended to confirm these findings, highlighting the need for further research before their widespread clinical application.

Our study has notable strengths; these include the utilization of a validated method for data collection through the use of SQ-FFQ [24], ensuring the reliability and accuracy of the gathered information. The involvement of well-trained personnel, adhering to standardized operating procedures, in the investigation of exposures further increases the robustness of our study. In addition, our GRS approach offers a more comprehensive understanding compared to relying on a single gene variant. The construction and utilization of a GRS enables a cumulative assessment of genetic predisposition, thereby enhancing our ability to understand the intricate associations between genetic factors and the disease [17]. However, our study has certain limitations. Despite adjusting our analysis for various factors, the potential for confounders caused by unmeasured or unknown variables exists [11]. This study has a relatively small sample size, but we were still able to identify a significant interaction, suggesting that our study has the statistical power to detect interactions. While food intake was recorded using a validated methodology, it is important to acknowledge the presence of measurement errors and recall bias in self-reported FFQs, which may introduce variability into the actual underlying interactions between the genetic and dietary factors affecting metabolic traits. Finally, it is essential to note that our study population was limited to Minangkabau women and cannot be generalized to the entire Indonesian population. Additionally, the absence of comparable studies for result benchmarking shows the need for future research to provide a more comprehensive understanding of the interplay between dietary and genetic factors and their impact on metabolic traits within diverse populations.

Our findings indicate the importance of promoting dietary habits that align with the recommended MUFA intake for the overall health and well-being of Minangkabau women, particularly those with genetic cardiometabolic risk. Improper dietary parameters seem to lead to the onset of metabolic diseases such as diabetes. Strategies to increase awareness and encourage the adoption of MUFA-rich foods may play a crucial role in addressing this nutritional shortfall among the study participants and potentially the Indonesian population.

## 5. Conclusions

The findings of our study show a significant interaction between a 23-SNP metabolic GRS and lifestyle factors, specifically impacting HbA1c levels. This study revealed a concerning inadequacy in dietary MUFA intake, potentially amplifying the genetic susceptibility of individuals to metabolic diseases. These results carry significant implications for public health, with the necessity of considering lifestyle factors alongside genetic predispositions. The lower MUFA intake observed emphasizes the importance of implementing targeted interventions to address nutritional gaps. However, future trials involving larger sample sizes are recommended in order to validate our findings before defining interventional dietary strategies for the Indonesian population. Understanding how genetic factors influence an individual’s response to dietary interventions could pave the way for personalized nutrition strategies that are tailored in order to mitigate the risk of metabolic diseases.

## Figures and Tables

**Table 1:** The characteristics of the study participants stratified based on their genetic risk score.

Variables	GRS Groups		*p* Value
	Low Risk (*n* = 53)	High Risk (*n* = 53)	
	Mean ± SD		
BMI (kg/m^2^)	1.4 ± 0.0	1.4 ± 0.0	0.084
WC (cm)	1.9 ± 0.2	1.9 ± 0.1	0.373
Fat Mass (kg)	1.3 ± 0.3	1.3 ± 0.1	0.074
Glucose (mg/dL)	1.9 ± 0.0	1.9 ± 0.0	0.642
HbA1c (ng/mL)	2.6 ± 0.3	2.7 ± 0.3	0.385
Fasting insulin (ml/UL)	4.4 ± 0.3	4.4 ± 0.2	0.380
Total Energy (Kcal)	3.3 ± 0.1	3.2 ± 1.3	0.040
Protein (g/day)	1.9 ± 0.2	1.8 ± 0.2	0.791
Fat (g/day)	1.8 ± 0.2	1.7 ± 0.2	0.206
Fiber (g/day)	0.9 ± 0.2	0.9 ± 0.2	0.375
SFA (g/day)	1.3 ± 0.2	1.2 ± 0.2	0.586
MUFA (g/day)	0.9 ± 0.2	0.8 ± 0.2	0.770
PUFA (g/day)	0.8 ± 0.2	0.7 ± 0.2	0.936
Physical Activity (min/week)	2.9 ± 0.5	2.9 ± 0.4	0.897

Data presented as means ± standard deviation (SD) for log transformed continuous variables. Low Risk—participants with ≤13 risk alleles; High Risk—participants with >13 risk alleles. Confounding factors include age, total energy (when a dietary factor is the outcome) and BMI (when BMI is not the outcome). Log transformation was applied to variables that were not normally distributed, including age (years), BMI (kg/m^2^), WC (cm), physical activity (min/week), fat mass (kg), glucose (mg/dL), cholesterol (mg/d), HDL (mg/dL), LDL (mg/dL), TGL (mg/dL), HbA1c (ng/mL), fasting insulin (mL/UL), total energy (kcal), protein (g/day), fat (g/day), fiber (g/day), SFAs (g/day), MUFAs (g/day), and PUFAs (g/day). BMI—Body Mass Index; WC—waist circumference; HbA1c—glycated hemoglobin; SFAs—saturated fatty acids; MUFAs—monounsaturated fatty acids; PUFAs—polyunsaturated fatty acids; GRS—Genetic Risk Score.

**Table 2:** Interaction between GRS and dietary factors on anthropometric and biochemical parameters.

	Protein (g/Day)	Fat (g/Day)	Fiber (g/Day)	SFA (g/Day)	MUFA (g/Day)	PUFA (g/Day)	Physical Activity (min/Week)
BMI (kg/m^2^)	0.907	0.590	0.290	0.948	0.858	0.961	0.819
WC (cm)	0.337	0.143	0.737	0.208	0.177	0.921	0.926
Fat Mass (kg)	0.769	0.863	0.270	0.713	0.917	0.652	0.626
Glucose (mg/dL)	0.302	0.259	0.762	0.379	0.165	0.414	0.366
Cholesterol (mg/dL)	0.277	0.327	0.158	0.627	0.386	0.339	0.753
HDL (mg/dL)	0.953	0.831	0.722	0.250	0.661	0.978	0.087
LDL (mg/dL)	0.791	0.841	0.387	0.957	0.581	0.821	0.215
TGL (mg/dL)	0.269	0.217	0.515	0.144	0.469	0.630	0.562
HbA1c (ng/mL)	0.526	0.376	0.132	0.225	0.026	0.127	0.936
Fasting Insulin (mL/UL)	0.844	0.809	0.985	0.576	0.172	0.211	0.623

Confounding factors include age, total energy (when a dietary factor is the outcome) and BMI (when BMI is not the outcome). BMI—Body Mass Index; WC—waist circumference; HDL—high-density lipoprotein; LDL—low-density lipoprotein; TGL—triglycerides; HbA1c—glycated hemoglobin; SFAs—saturated fatty acids; MUFAs—monounsaturated fatty acids; PUFAs—polyunsaturated fatty acids; GRS—Genetic Risk Score. *p*-value for significant interactions between GRS and lifestyle factors on anthropometric and biochemical parameters was calculated using a linear regression analysis.

## Data Availability

The original contributions presented in this study are included in the manuscript. Further inquiries can be directed to the corresponding author.

## Supplementary Materials

The following supporting information can be downloaded at: https://www.mdpi.com/article/10.3390/nu16173022/s1, Table S1: Genotype distribution of 23 SNPs among 106 Minangkabau women.
