# Supplementary material for: A Novel Interaction between a 23-SNP Genetic Risk Score and Monounsaturated Fatty Acid Intake on HbA1c Levels in Southeast Asian Women
Source: Nutrients. 2024 Sep 6;16(17):3022. doi: 10.3390/nu16173022 (PMC11397529; doi:10.3390/nu16173022)
Supplement: Supplementary file 1 [file nutrients-16-03022-s001.zip › nutrients-3151810-supplementary.pdf]

Table S1. Genotype distribution of 23 SNPs among 106 Minangkabau women

Gene	SNP	Major/Minor Allele	Effect Allele	Major/Minor Frequency (n)	Allele Frequency (%)	Genotype Frequency (11/12/22 in n)	Genotype Frequency (11/12/22 in %)	HWE
MTHFR	rs1801133	C/T	T	192/20	90.57/9.43	86/20/0	81.1/18.9/0	0.283
TCF7L2	rs7903146	C/T	T	194/18	91.51/8.49	88/18/0	83/17/0	0.339
FTO	rs8050136	C/A	A	162/50	76.42/23.58	62/38/6	58.5/35.8/5.7	0.955
MC4R	rs17782313	T/C	C	185/27	87.26/12.74	80/25/1	75.5/23.6/0.9	0.529
TCF7L2	rs12255372	G/T	T	195/17	91.98/8.02	89/17/0	84/16/0	0.369
PPARG	rs1801282	C/G	G	200/12	94.34/5.66	94/12/0	88.7/11.3/0	0.536
KCNJ11	rs5219	C/T	T	143/69	67.45/32.55	51/41/14	48.1/38.7/13.2	0.220
FTO	rs9939609	T/A	A	162/50	76.42/23.58	62/38/6	58.5/35.8/5.7	0.955
KCNQ1	rs2237895	A/C	C	150/62	70.75/29.72	54/42/10	50.9/39.6/9.4	0.661
CYP2R1	rs10741657	G/A	A	110/102	51.89/48.11	31/48/27	29.2/45.3/25.5	0.338
NADSYN1	rs12785878	G/T	G	44/168	20.75/79.25	7/30/69	6.6/28.3/65.1	0.150
CYP24A1	rs6013897	T/A	A	140/72	66.04/33.96	48/44/14	45.3/41.5/13.2	0.442
GC	rs2282679	A/C	C	172/40	81.13/18.87	71/30/5	67/28.3/4.7	0.436
CYP2R1	rs12794714	G/A	A	158/54	74.53/25.47	57/44/5	53.8/41.5/4.7	0.336
DAB1	rs6680429	A/G	A	97/115	45.75/54.25	21/55/30	19.8/51.9/28.3	0.641
ADIPOQ	rs266729	C/G	G	138/74	65.09/34.91	43/52/11	40.6/49.1/10.4	0.412
KCNQ1	rs2237892	C/T	T	130/82	61.32/38.68	42/46/18	39.6/43.4/17.0	0.380
CDKN2A/B	rs10811661	T/C	T	70/142	66.98/33.02	9/52/45	8.5/49.1/42.5	0.261
CASR	rs1801725	G/T	T	191/21	90.09/9.91	87/17/2	82.1/16/1.9	0.296
CAPN10	rs5030952	C/T	T	169/43	79.72/20.28	70/29/7	66/27.4/6.6	0.112
ABCD4	rs3742801	G/A	T	146/66	68.87/31.13	51/44/11	48.1/41.5/10.4	0.742
MMAA	rs2270655	G/C	C	198/14	93.40/6.60	92/14/0	0/13.2/86.8	0.466
FUT6	rs778805	T/C	A	98/114	46.23/53.77	21/56/29	19.8/52.8/27.4	0.518

[1]SNP-Single nucleotide polymorphism; [2]HWE-Hardy Weinberg Equilibrium, estimated using Chi-Squared analysis (X^2)
